# Microfluidic 3D Helix Mixers

**DOI:** 10.3390/mi7100189

**Published:** 2016-10-17

**Authors:** Georgette B. Salieb-Beugelaar, Daniel Gonçalves, Marc P. Wolf, Patrick Hunziker

**Affiliations:** 1 Nanomedicine Research Lab CLINAM, University Hospital Basel, Bernoullistrasse 20, CH-4056 Basel, Switzerland; daniel.goncalves@swissnano.org (D.G.); marc.wolf@usb.ch (M.P.W.); patrick.hunziker@swissnano.org (P.H.); 2 The European Foundation for Clinical Nanomedicine (CLINAM), Alemannengasse 12, CH-4016 Basel, Switzerland; 3 Intensive Care Clinic, University Hospital Basel, Petersgraben 4, CH-4031 Basel, Switzerland

**Keywords:** circular helical mixers, subtractive microfabrication, polydimethylsiloxane (PDMS), nanomaterials, nanoparticles, nanomedicine

## Abstract

Polymeric microfluidic systems are well suited for miniaturized devices with complex functionality, and rapid prototyping methods for 3D microfluidic structures are increasingly used. Mixing at the microscale and performing chemical reactions at the microscale are important applications of such systems and we therefore explored feasibility, mixing characteristics and the ability to control a chemical reaction in helical 3D channels produced by the emerging thread template method. Mixing at the microscale is challenging because channel size reduction for improving solute diffusion comes at the price of a reduced Reynolds number that induces a strictly laminar flow regime and abolishes turbulence that would be desired for improved mixing. Microfluidic 3D helix mixers were rapidly prototyped in polydimethylsiloxane (PDMS) using low-surface energy polymeric threads, twisted to form 2-channel and 3-channel helices. Structure and flow characteristics were assessed experimentally by microscopy, hydraulic measurements and chromogenic reaction, and were modeled by computational fluid dynamics. We found that helical 3D microfluidic systems produced by thread templating allow rapid prototyping, can be used for mixing and for controlled chemical reaction with two or three reaction partners at the microscale. Compared to the conventional T-shaped microfluidic system used as a control device, enhanced mixing and faster chemical reaction was found to occur due to the combination of diffusive mixing in small channels and flow folding due to the 3D helix shape. Thus, microfluidic 3D helix mixers can be rapidly prototyped using the thread template method and are an attractive and competitive method for fluid mixing and chemical reactions at the microscale.

## 1. Introduction

Microfluidic systems are of growing importance for applications in the field of, for example, nanomedicine, biomedicine, rapid diagnostics and single cell investigations [1,2,3,4]. The production of microdevices developed rapidly over the last decade and commonly used methods are standard photolithography, micromachining and polymer molding. The advances in 3D printing techniques offer new possibilities for the manufacturing of microdevices such as the use of liquid metals as fugitive inks for the manufacturing of channels [5], the production of leak free seals [6] or the use of fused filament to produce devices with circular channels. For further reading, see for example Ho et al. [7].

**Figure 1:**
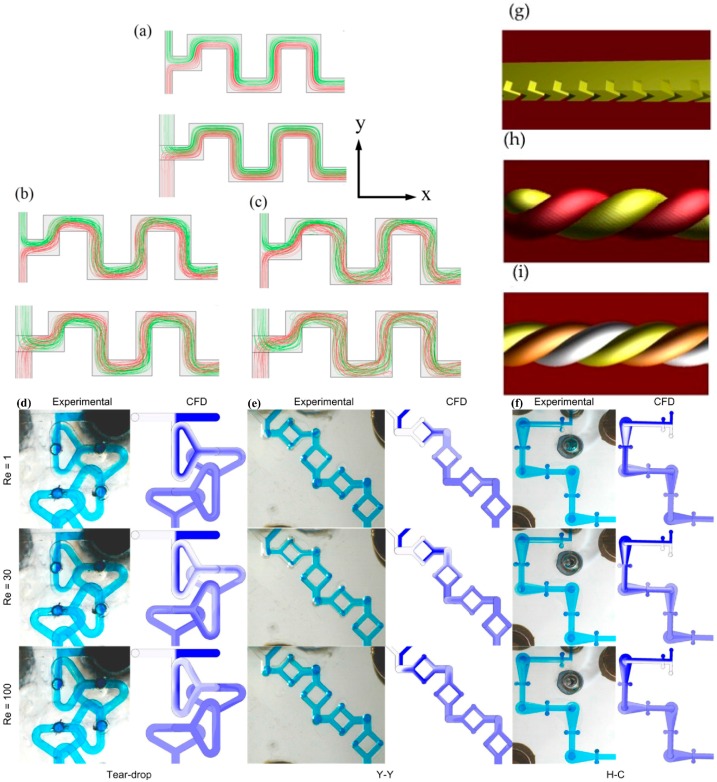
Various mixing geometries. (**a**–**c**) Two flows (**green** and **red**) in two different sizes of serpentine type of microchannels at *Re* = 0.1 (**a**), 30 (**b**) and 90 (**c**) (with permission from Shakhawat Hossain [10]). Other geometries are presented in (**d**) the Teardrop, (**e**) the Y–Y and (**f**) the H–C mixing geometries at *Re* = 1 (**top**), 30 (**middle**) and 100 (**bottom**). The **left** column of each geometry represents an experiment, where as on the **right** the images taken from computer simulations (with permission from Vladimir Viktorov [11]). (**g**) The herringbone mixer, (**h**) a double helix and (**i**) a triple helix mixer. The latter two are part of the work presented in this manuscript.

The mixing of fluids in microfluidic devices is an important application and has received considerable attention recently. A key challenge in mixing is to utilize solute diffusion as well as possible, and at the same time optimize convective contributions to fluid mixing. This is a design challenge because diffusion occurs best in very narrow channels that imply, on the other hand, low Reynolds numbers (<<2000) that force the physics of flow towards the laminar regime and suppress turbulence. Important additional design considerations include simplicity of design, reproducibility and availability of rapid prototyping methods. Micromixers can be classified into passive and active mixers. In a passive micromixer, solutions inside a microchannel are mixed by enhancing the diffusion mixing component by suitably equipping a microchannel with additional features to exploit inertial forces, chaotic effects and flow folding. In contrast, in active mixers, an external force is applied to the fluid for mixing, such as an acoustic force [8] or magnetic force [9]. An advantage of active mixing is that channel length can be reduced in comparison to a passive mixer; however a disadvantage might be the need of additional external instruments. In this work, recent advances in rapid prototyping for true 3D channel geometries in polymeric microfluidic systems are utilized to improve passive mixing. Examples of mixing geometries are discussed below and presented in Figure 1.

Liu et al. [12] investigated mixing by using a serpentine shaped microchannel for Reynolds numbers 6–70 and found that such geometries are able to disturb the laminar flow at these Reynold numbers, similar to the events found in chaotic advection at higher Reynolds numbers and in larger channels.

Chen and Meiners [13] presented topologic mixing by using helical channels that repeatedly fold the flow resulting in an exponential increase of the concentration gradients and with this, they obtained fast and efficient mixing by diffusion at Reynolds numbers between 0.1–2. Kumar et al. [14] studied curved channels and concluded that for higher Schmidt number fluids, the mixing is improved at *Re* ~ 10, however is not affected at *Re* ~ 0.1. The Schmidt number (*Sc*) is a dimensionless number defined as the ratio of viscosity and mass diffusivity. Additionally, it is shown that at low values of curvature ratio, the mixing is higher as compared to a higher curvature ratio. Lee et al. [15] developed a split and recombination micromixer (also called a “splitting and recombination” (SAR) mixer). Here, structured units in a microchannel were used to increase the number of interfaces, which enhances mixing. They showed that mixing could be completed for 90% after 7 units and at a Reynolds number of 0.6. The experimental results used for quantifying the mixing efficiency are in agreement with numerical analysis.

A novel generation of this 3D SAR micromixer (called chain micromixer) was developed by Viktorov and Nimafar [16] where the mixing performance of using microstructures on both the top and bottom floors of the microchannels were compared with other micromixers (e.g., T mixers). It was shown by both experimental as well as computational fluid dynamics that these chain mixers in particular have an efficacy of up to 98% that can be achieved at Reynolds numbers from 0.083 ≤ *Re* ≤ 4.166. Another study showed that mixing is also obtained by unbalanced splits and collisions of fluid streams and Dean vortices [17] at *Re* numbers from 10–80. Here the highest mixing performance was obtained by *Re* > 40. The structures used could be described as a main channel, which is split into two sub-channels that are unequal in width and recombine after a certain distance. The repetition of such units would enhance mixing. Lim et al. [18] designed a 3D crossing manifold micromixer, for which it was estimated by numerical simulation that a mixing ratio of 90% could be obtained for a channel length that is five times shorter than the channel width (*Re* ~ 1; Péclet ~ 1000). The Péclet number is a dimensionless number defined to be the ratio of the rate of advection of a physical quantity by the flow, to the rate of diffusion.

The fabrication of 3D microchannel systems in polydimethylsiloxane (PDMS) is in most cases a multilayer process as for example presented by Wu et al. [19]. They developed a method that uses planar structures or pseudo 3D structures to generate real 3D geometry in a microfluidic system. The pseudo 3D structures are planar sheets of channels, which are bent into 3D geometries and subsequently embedded in PDMS to freeze the geometries. The manufacturing of 3D circular channel networks remains a challenge but progresses rapidly due to advances in 3D template casting methods that allow design of complex, connected 3D geometries with programmable flow exhibiting various channel geometries [20]. The progress in 3D printing techniques are shown by the work of Hwang et al. [21] who used a 3D printed mold to produce cylindrical microfluidic channels ranging from 200–1000 μm and compared these results with four different 3D printers. Parker et al. used a 3D printer with 16 μm layer resolution to manufacture a device capable of creating 200 μm hydrogel droplets [22]. Other manufacturing methods are presented by, for example, Song et al. [23] who used a metal wire (solder) process to produce helical channels into PDMS. By heating up the polymerized PDMS to 190 °C, the solder could be removed. Verma et al. [24,25] showed that nylon thread could be formed into 2D or 3D structures and used as templates to generate helical channels.

Complex connectivity and geometries are relevant for various biomedical, chemical and pharmacological applications. Here, circular channel networks remain, due to their geometry, of interest for biomedical applications such as for the study of the rheological behavior of blood cells in microcirculatory blood flows. Still, a major part of these investigations is done by modeling. Further development of the models will require validation where the availability of circular microfluidic structures or networks might serve as an alternative to in vivo experiments (e.g., [26]). A final and important example is the generation of nanoparticles based on polymers with these microsystems, which are attractive due to their flexibility in design, synthesis and functionalization. Such nanoparticles are an enabling technology for drug delivery and receptor-targeting required in the personalized medicine of the future. Our reports on the biomedical usage of such intelligent nanomaterials were a key trigger to develop improved assembly methods [27,28]. Here, one of the key advantages is the ability, in principle, to tune the size and shape of the nanoparticles, which can critically influence behavior (e.g., stability, delivery, elimination time) in vivo. Micelles for example, consist of hydrophilic shells and hydrophobic cores, that are well suited for the encapsulation of hydrophobic molecules, such as drugs, diagnostic agents, or functional materials, and poly(2-methyl-2-oxazoline)–polydimethylsiloxane (PMOXA–PDMS) nanosystems in particular are known for biocompatibility, stealth properties and low permeability.

In this study, we further extended the recently published method of complex thread template casting [20] to develop a new type of true 3D circular helical micromixers. The potential of such micromixers is explored by hydraulic analysis and fluid dynamics modeling and is experimentally tested by using the systems for multicomponent chemical reaction and nanomaterial assembly of advanced nanomaterial-forming polymers [29].

## 2. Experimental Section

### 2.1. Thread Helices and PDMS Cast

The manufacturing method was used as described previously [20]. In brief, mono/multi low-surface-energy threads with diameters of 120 and 200 μm made from polyamide and copolymer (Vexter pro-type, Nikko, Japan; Fighter Fluorostrong 120 and 180 μm and Stroft abrasion resistance (ABR), silicon-polytetrafluoroethylene (PFTE) tempered Monofil 200 μm) were used to manufacture structures to be cast in PDMS. Access ports were done using medical Luer-port needles. The needles used were: BD Microlane 3 (30 G × 1/2 inch, 0.3 mm × 13 mm, BD, Drogheda, Ireland), Terumo Neolus (25 G × 5/8 inch (0.5 mm × 16 mm) and 20 G × 1-1/2 inches (0.9 mm × 40 mm), Terumo Europe, Leuven, Belgium). Falcon^®^ Integrid Petri dishes served as structural elements (Taylor Scientific, St Louis, MO, USA). Express Silicon (Pattex; Henkel Central, Wien, Austria) was used to close the holes, to avoid PDMS leakage, prior casting. Holes were drilled into the walls of the Petri dishes to allow Luer needles to be used as access ports for the interconnected 3D networks. Through the needles, the threads are passed through and helical networks could be generated, serving as a template, where the close thread contact will result in a connection in between the channels upon thread removal after curing the PDMS (Sylgard 184, Dow Corning, Midland, MI, USA). A Pean forceps (Peha-instrument; Hartmann, Heidenheim, Germany) was used to remove the threads of the PDMS devices.

**Figure 2:**
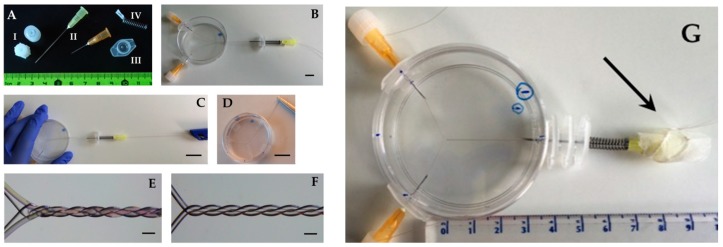
Manufacturing and reproducibility. (**A**) I: Example of caps that were used to fix the threads (see (**B**)), II: needles, III: Eppendorf caps that were cut off the tubes and IV: a ballpoint spring (120 N/m) used to obtain pressure on the thread helix. (**B**) Through the holes that were made in the wall of the Petri dish, the needles and the threads were inserted, followed by immobilization of the two threads as presented in (**C**). (**C**) A helix structure in the threads was generated by twisting and gently pulling the threads (**A**); a spring (IV) serves to exert a defined pulling force on the filament and needle (closed by a cap) during polymerization (**A**-I and **G**, black arrow). Holes in the Petri dish were closed with a drop of polydimethylsiloxane (PDMS) or Express Silicon. (**D**) After polymerization, caps were removed to release the threads. Excess length of threads was cut (only their orange needle ends). Needles were removed carefully and the threads were slowly removed using Pean forceps. (**E**) Thread is still in the channel. (**F**) Channel after removal. (**G**) The pitch size can be controlled by adapting the twist count prior to casting. Turning the yellow needle around allows to increase/decrease the pitch number/length (black arrow). The bars represent in (**B**) 1 cm, (**C**,**D**) 2.5 cm and (**E**,**F**) 400 μm.

**Figure 3:**
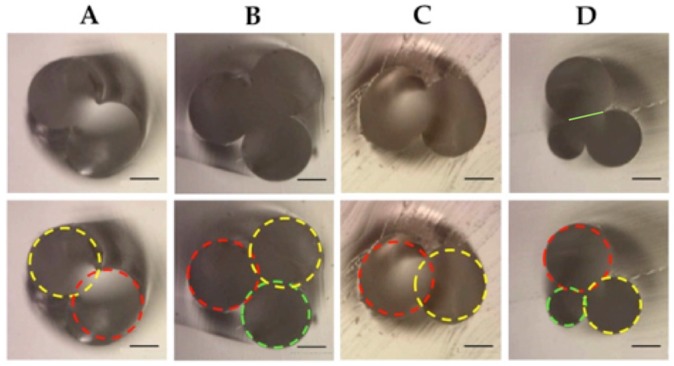
(**A**,**B**) are cross sections from design I (Figure 4). (**A**) A cross section of a double helix made by using two threads with a diameter of 200 μm. (**B**) A triple helix made with two threads of 200 μm and one of 180 μm in diameter (green circle in bottom image). (**C**) A double helix (design II; Figure 4) made by using two threads with diameter of 200 μm; and (**D**) (design III; Figure 4), a triple helix where three different thread diameters were used: 120, 180, and 200 μm. The bar represents 100 μm.

**Figure 4:**
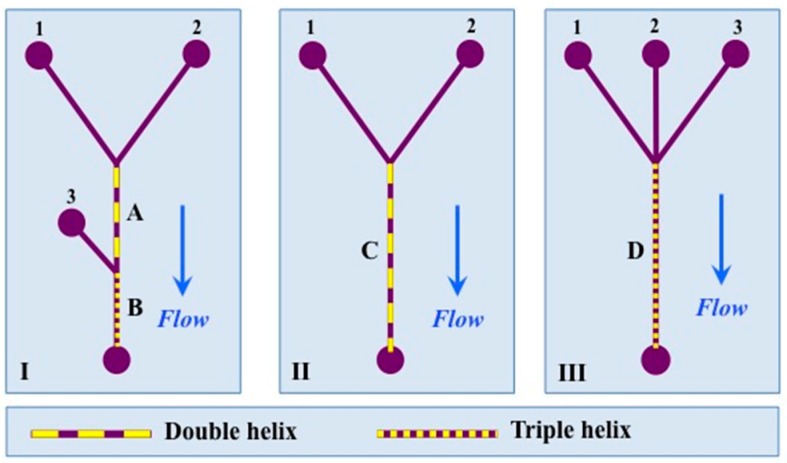
A schematic presentation of the three designs used in this work (not to scale).

Figure 2 presents materials and manufacturing steps of a double helix. We found that introducing threads through fine needles is no major challenge, and that thread withdrawal is reproducibly achieved with a little training if the design does not consist of too long channels with too narrow filaments. A 2 cm channel length with helix structure and thread diameters reported here can be achieved. A triple helix can be produced in similar way by adding an additional thread. This method proved to be simple and reproducible with little training. Pitch length can be calculated and adapted prior casting in PDMS as was done for the device in Figure 2 (produced in triplicate). The final pitch length of this device was between 1.41–1.48 mm per turn and the final channel length ~22 mm. PDMS was mixed in a 10:1 ratio according to the manufacturer’s recommendation, was subjected to vacuum conditions for 20 min to remove bubbles, followed by curing at 60 °C, overnight. In addition to the reproducibility of the pitch and channel length, the diameters of the helices were also reproducible. Figure 3 shows examples of cross sections of designs that were used in this work (Figure 4). The circles drown in each image in bottom row represent the thread diameter. The images and the evident slight overlap of the fitting circles document that two neighboring filaments create a conformal contact zone devoid of PDMS that serves later as the fluid connection between two channels. This conformal contact zone is determined by: (a) transverse elastic properties of the filament; (b) curvature of the filament; (c) force applied to the filament. In our experience, the dimension of this conformal contact zone is reproducible within a device and between devices when the mentioned three design parameters are kept constant.

Reproducibility of channel connectivity was determined visually and quantitatively by serial cross sectioning. We found a mean diameter of the channel connection (green line in Figure 3D top) was 110 (SD 6) μm, and 109 (SD 3) μm respectively, measured five-fold in two separate devices, thus confirming good conformal contact and good reproducibility of the channel communication geometry.

**Figure 5:**
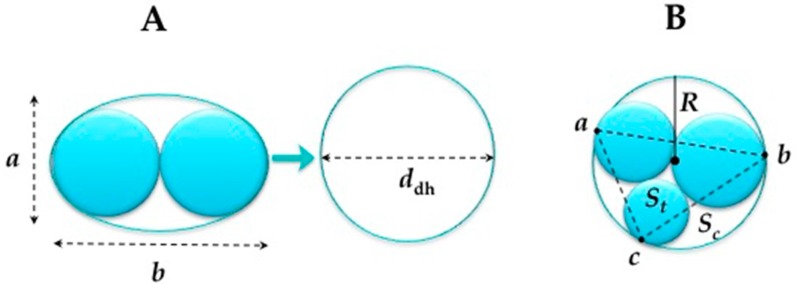
(**A**) The equivalent circular diameter calculated for the double helix (*d*_dh_). (**B**) Heron’s Formula used to calculate the radius, *R*, of the circumcircle used as an approximate radius of the triple helix.

According to the type of helix, a representing diameter/radius was calculated by using the images and at least two separate devices of the same design (see Section 2.4 Helix Characteristics and Figure 5).

### 2.2. Imaging

Macroscopic and microscopic images were acquired using an Apple iPhone camera (8 megapixels, Apple, Cupertino, CA, USA) and a Dino-Lite microscope camera (5 megapixels, AnMo Electronics Corporation, Hsinchu, Taiwan) and were processed using the ImageJ software (version 1.45s, National Institutes of Health, New York, NY, USA) [30].

### 2.3. Pressure Drop Along the Microchannels

The Hagen–Poiseuille’s law gives the pressure drop in an incompressible Newtonian fluid through a long cylindrical pipe with a constant cross section that is substantially longer than its diameter:

(1) $$Q=\frac{\pi R^{4}\Delta P}{8\eta L}$$

where *Q* is the volume flow rate (m^3^/s), *R* is the radius (m), Δ*P* the pressure difference (Pa), η the viscosity (Pa·s) and *L* the length of the channel (m). In addition, it is assumed that there is a constant laminar flow and that the helical channel structures act as laminar mixers. The atmospheric pressure was used to calculate the pressure drop along the channels in the devices next to the experiments with known volume flow rates.

### 2.4. Helix Characteristics

(1) For the double helices the equivalent circular diameter was calculated using the following equation (see Figure 5A):
  *d*_dh_ = 1.55 *A*^0.625^/*P*^0.25^ (2)
  where *A* = the cross-sectional area of the oval (m^2^); = π*ab*/4 (*a* = major dimension of oval, *b* = minor dimension oval) and
  *P* = approximate perimeter of oval (m) = 2π (1/2 ((*a*/2)^2^ + (*b*/2)^2^))^1/2^ (3)
(2) When the radii of the threads are all unequal as presented in Figure 5B, the radius is estimated by the circumcircle radius using Heron’s formula.
  (4) $$R\;=\;\frac{abc}{4\;\sqrt{s\left(s-a\right)\left(s-b\right)\left(s-c\right)}}$$
  where *s* = (*a* + *b* + *c*)/2, the triangle area $St=\sqrt{s\left(s-a\right)\left(s-b\right)\left(s-c\right)}$ and the circumcircle area *Sc* = πR^2^.
(3) The Reynolds number (*Re*)
  *Re* = (ρ*Vd*)/η (5)
  where ρ is the density of the fluid (kg/m^3^), η the kinematic viscosity (m^2^/s). The kinematic viscosity for water at room temperature is 1.004 m^2^/s.
(4) The Schmidt number (*Sc*)
  *Sc* = η/*D* = μ/(ρ*D*) (6)
  where, *D* is mass diffusivity (m^2^/s), μ the dynamic viscosity of the fluid (Pa·s or N·s/m^2^ or kg/m·s) and ρ is the density of the fluid (kg/m^3^). *Sc* is assumed to be 340 for a dilute solution in water.
(5) The Péclet number (*Pe*)
  *Pe* = *Sc* × *Re* (7)
(6) The radius of curvature of the helices (*Rc*) is larger than the radius of the channel (*R*) leading to the secondary Dean flow, which is due to the inhomogeneous flow inside the helices and is quantified by the Dean number (*De*).
  *De* ~ *Re* (*d*/2*Rc*)^1/2^ (8)
  where *Re* = the Reynolds number, *d* = the diameter of the helix and *Rc* = the radius of curvature.
  *Rc* = 1/curvature (9)
  where
curvature = *R*/*R*^2^ + *c*^2^ (10)
  and
  *c* = total length/2π × total number of pitches. (11)
  Related to this is the curvature ratio λ = *Rc*/*R* used by others to study the mixing performance (e.g., [14]).
(7) For a circular channel with diameter *d* (μm), the time required for mixing due to molecular diffusion in a laminar flow across the channel is approximately: *t*_mix_ (s) = *d*^2^/*D*. The diffusion coefficient *D* is known to range from 9.3 × 10^−9^ m^2^/s at 300 K for protons (including water), to 2.3 × 10^−9^ m^2^/s for water, to ~4 × 10^−10^ m^2^/s for small molecular dyes, to ~4 × 10^−12^ m^2^/s for 100 nm particles. The length required for mixing due to molecular diffusion in a laminar flow *L*_mix_ (m) = *t*_mix_ × *V*, with the average velocity in *V* (m/s).
(8) The equation (diffusion equation) for incompressible flow
  (12) $$\frac{\partial \varepsilon }{\partial t}=D\nabla^{2}\varepsilon -V\times \nabla \varepsilon$$
  where ε is the variable of interest and *V* the average velocity of the fluid was used.

### 2.5. Microchannel Chemical Reaction

As model chemical reaction, we chose the pH-induced color change of the pH-indicator Phenol Red. The color change allows optical monitoring, and the high reaction speed allows separating mixing kinetics from chemical reaction kinetics. The ability to run the reaction with two or three reaction components (phenol red, sodium hydroxide, hydrochloric acid) allows testing 2-channel and 3-channel designs. Phenol-red (Fluka AG, Buchs, Switzerland), NaOH and HCl were used at 2 mM. A pH indicator dye is a suited approach to determine mixing degree, because unmixed fluid elements are clearly evident by their color. While this could be done automatically using quantitative spectral image analysis, we found that standard RGB video still frames of the channel flow yielded clear results, too. This approach also allows to detect multistage mixing, as shown in this work. pH-triggered, color-based determination of mixing thus appears to be a simple but effective tool.

### 2.6. Assembly of Nanoparticles

The model polymer used is based on poly(2-methyl-2-oxazoline)-block-poly(dimethylsilox-ane)-block-poly(2-methyl-2-oxazoline) with a hydroxyl functional group, represented by OH-PMOXA_12_-PDMS_73_-PMOXA_12_-OH [27,28,31,32].

A solution of 20 mg/mL of already synthesized polymer in ethanol was prepared in a 1 mL syringe, and 1× PBS buffer was prepared in a 20 mL syringe to serve as aqueous phase. Both syringes were systemized in electric syringe pumps (Perfusor**^®^** Compact, B|Braun Medical AG, Sempach, Switzerland) and connected to the microfluidic device through tubing as described earlier [29]. Two independent runs were designed with variation of two parameters (see Table 1): flow rate ratio (FRR = *Q*_aq_/*Q*_org_), and total flow rate (*Q_T_* = *Q*_aq_ + *Q*_org_), known to influence nanoparticle formation and size distribution in these systems [29]. From each run, two consecutive samples of approximately 500 μL were recovered. No leakage was found in the system during the whole experiment. Nanoparticle formation and sample size distribution was accessed by Dynamic light scattering (DLS) measurements (directly from the microfluidic assembly at 25 °C), and performed on a Malvern Zetasizer Nano S (ZEN 1600; Malvern, Herrenberg, Germany). The distribution curve was calculated with a CONTIN fit for random distribution. Cumulant analysis was performed on the correlation function, and mean intensity was collected for static light scattering interpretation.

### 2.7. Computational Modeling

Helical channels were modeled using our Oberon Volume Modeling software, fluid dynamics modeling was performed using our Oberon MultiPhysics software, and visualization was done using our Volume Raytracer package (written by P.H., based on MathOberon (ETH-Oberon, Zürich, Switzerland) [33], see also http://www.computational.ch). With a channel diameter of *r*, the helix extending along the *z*-axis and obeying the structure formula *x* = *qr*sin(4*z*π), *y* = −*qr* + *qr*cos(4*z*π) for the center line of each component channel was created. A *q* = 0.9 was chosen to induce a partial overlap of each component channel that is required for mixing and that, in the physical design, is introduced by the conformal contact of the polymeric thread.

The incompressible Navier–Stokes equation with convection and diffusion was modeled. Pressure driven inflow and outflow conditions and non-slip channel boundaries were used. Fluid dynamics modeling was performed in a multiresolution fashion with the finest finite difference grid step size of 4 μm, a time step of 3 μs, and a finest grid point count of >1,000,000. A first-order implicit Euler scheme was used for time stepping. Physical fluid characteristics for water and a driving pressure of 1.25 kPa per millimeter channel length were chosen. Simulation was run until steady state flow characteristics were reached. Mixing efficiency can be determined in simulation by planar maximum-intensity and mean-intensity projections of the solute concentration in the fluid column, because the maximum intensity decays linearly with mixing, and mean-intensity over a channel shows spatial distribution of the solute.

## 3. Results and Discussion

### 3.1. Mixing

**Figure 6:**
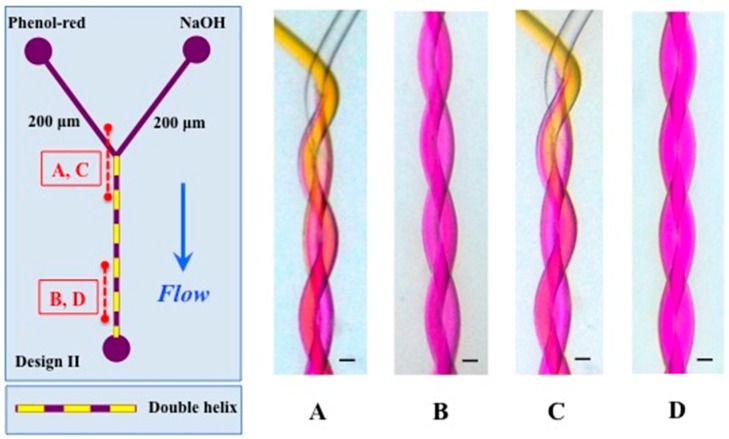
Design II on the **left** shows positions of (**A**–**D**). (**A**,**C**) present the inlet and, in (**B**,**D**), mixing is clearly observed. The conditions of (**A**,**B**) were: NaOH (2 mM) and Phenol-red (2 mM) 2 mL/h and of (**C**,**D**) NaOH (2 mM) and Phenol-red (2 mM) 0.5 mL/h. The channels of the double helix are 200 μm and the total length of the helix is 28 mm. The pitch length is 2.24 mm. The scale represents 200 μm. Channel segments shown in (**A**–**D**) have a length of 4.5 mm.

Mixing was first studied in a simple 2-channel design containing two 200 μm channels and having a helix length of 28 mm with a pitch length of 2.24 mm (Figure 6) using the color change of a pH dye as marker of mixing. Mixing is visible as a color change of the pH indicator and in this design is evident already after the first pitch. This is in strong contrast to the T-shaped device shown in Figure 9, where full mixing is not observed at the end of the channel.

**Figure 7:**
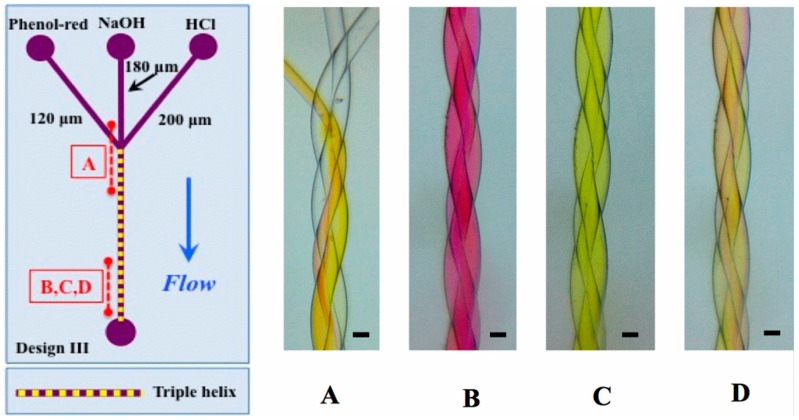
**Left**: channel design of type III. In (**A**–**C**): HCl, NaOH are at 2 mM, Phenol red 2 mM, 2 mL/h. Channel diameters in the triple helix are 120, 180, and 200 μm. Pitch length is 2.5 mm. Scale bar 200 μm. (**A**,**C**): inlet in acid-dominated regime (HCl at 2 mL/h, NaOH at 0.5 mL/h). (**B**): base-dominated regime (HCl at 0.5 mL/h, NaOH at 2 mL/h). (**D**): balanced acid-base regime, all flows at 1.5 mL/h. Mixing is evident at the top of (**B**–**D**) corresponding to a position of 15 mm after channel joint. Note the significant difference in mixing compared to Figure 9 with straight channels.

Next, a triple helix design was constructed and tested and is shown in Figure 7. Here, a differential diameter of the threads was chosen, giving additional flexibility to the design in situations where this might be desirable: for example, the flow resistance changes strongly with the channel diameter (Poiseuille law) and allows to tailor flow characteristics over a large range by simply changing the filament diameter. Three-component mixing was tested using hydrochloric acid, sodium hydroxide, and a pH indicator dye. Changing the relative flow between the channels led to consistently good mixing between the channels, but also along the channel, implying that the mixing process occurs continuously in time, rather than being a process with random fluctuations at the microscopic scale, suggesting that at this examined scale, mixing is not a chaotic process. This finding is also supported by the steady state flow and mixing that is found in the fluid dynamics simulation below.

**Figure 8:**
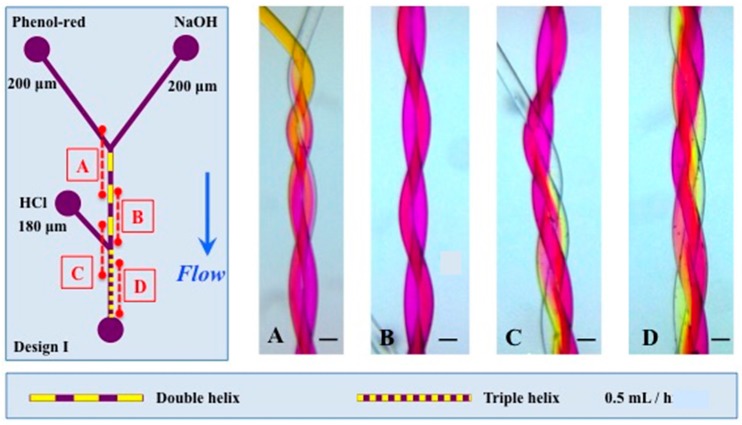
The left panel shows the design I, with the position of panels (**A**–**D**) along the channel. Channel lengths were 11 and 7 mm for the double and triple helix respectively. The channels of the double helix are 200 μm and the channel entering in the triple helix is 180 μm. The pitch length is 2.44 mm and 2.80 mm of the double and triple helix respectively. The scale represents 200 μm. The flow used was 0.5 mL/h for Phenol-red (2 mM), HCl (2mM) and NaOH (2 mM).

As a next step in design complexity, a multi-phase mixing device was constructed by having a double helix design in a first segment, followed by an additional side channel after a given distance as shown in Figure 8. This will allow to perform reactions sequentially in a channel without need for change of connectivity. In a first step, a pH indicator is mixed with hydroxide, showing rapid mixing as above. In a second step, the side channel adds acid to the mix, here in exact molar equilibrium with the base (thus creating a very fragile system sensitive to confounding factors). This now shows that over 7 mm, mixing is not complete in the final triple helix. Reasons for this finding might include the fact that the Dean number is very low in this system, in particular if such low flows are used; it might also be caused by a suboptimal conformal contact between the channels, although Figure 3 showing the cross section of the channels documents clearly that this was not the problem in this case. Mixing might be sped up by increasing the Dean number of the design, which is a function of the Reynold number (including velocity), the helix radius and helix curvature. Thus, increasing flow velocity and decreasing pitch length will increase mixing. Although increasing channel diameter would also increase the Dean number, which is not desirable here because of its strong negative effect on the diffusive component of mixing.

**Figure 9:**
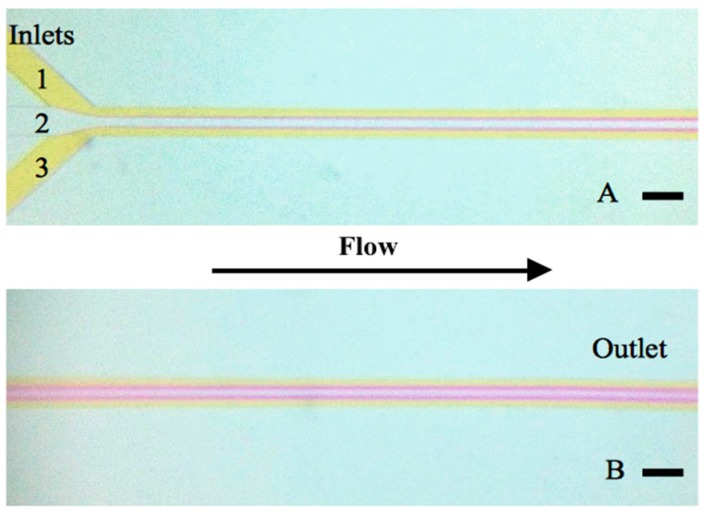
Conventional, planar microfluidic T-shape device with rectangular cross section used for nanoparticle self-assembly in [29]. The width is 200 μm, the height is 45 μm and the length from channel joint (**top left**) to outlet (**bottom right**) is 15 mm. Note the very slow diffusive mixing from inlet to outlet, evidenced by a maintained color difference even at the outlet of the device. Flow rates of 1 mL/h Phenol red (inlet **1**, **3**) and 0.5 mL/h NaOH (inlet **2**) are used. The scale represents 200 μm. Note that due to laminar flow characteristics, mixing occurs only slowly along the channels.

As a next step, the T-shaped, rectangular cross section microfluidic device reported in [29] for nanoparticle assembly, was used as a comparator device and is shown in Figure 9. The length of the channel was 15 mm, the width 200 μm, the height 45 μm and flow rates of 1 mL/h Phenol red (inlet 1, 3) and 0.5 mL/h NaOH (inlet 2) are used. The flow in this device was also laminar in the experiment as expected from the similarly low Reynolds number regime as above and as predicted by fluid dynamics simulation (not shown). Mixing was very slow in comparison with the helix devices. Note that the diffusion regime used here is the best that can be achieved practically, because the diffusion coefficient of protons that drives the color change is at least an order of magnitude larger than for most other reactants; in other chemical reactions, mixing would thus proceed even slower.

In these rectangular channels, one dimension is similar to the larger threads used above, while the other dimension is smaller, with a smaller overall cross section of the T-channel compared to the other geometries. Similar overall flows in a channel with narrower cross section will result in shorter transit time. Although it is generally difficult or impossible to achieve full dynamic similarity in the fluid dynamics sense in such dissimilar geometries, in particular when Navier–Stokes is coupled with convection–diffusion, the comparison done here is nevertheless instructive: The smaller dimension of the T device used for comparison favors mixing performance even with shorter transit time, because, since the seminal work of Albert Einstein in 1905 [34], it has been known that diffusion is inversely proportional to the square of distance but proportional to linear time; for a given (laminar) flow, a smaller cross section will thus favor mixing despite faster transit. Also, as driving pressure scales with the fourth power of diameter (Hagen–Poiseuille), good mixing in larger diameter channels may be of considerable interest for real-world applications because of the strongly relaxed requirements for driving pressure and mechanical device stability. From a practical viewpoint, a user would like to mix a given volume per time of fluid with a given maximum permissible driving pressure. Thus, the findings support the benefit of the new helix mixer design.

**Figure 10:**
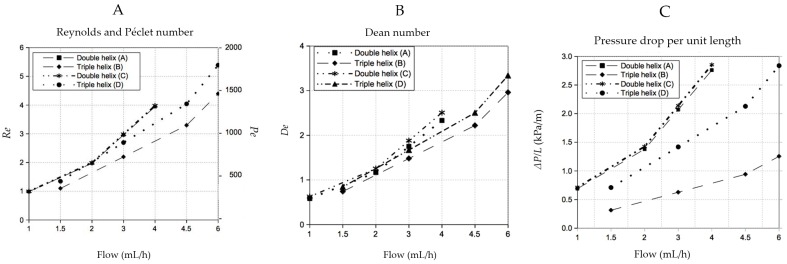
(**A**) Reynolds and Péclet numbers inside double and triple helices; (**B**) the Dean number and (**C**) the pressure drop/unit length along double and triple helices.

Figure 10 shows the Reynolds and Péclet, Dean numbers and the pressure drop Δ*p* encountered in the experiments. Table 2, presents characteristics of the helical channels as the calculated radius, radius of curvature, pitch length (one helical period where a full turn is made), and the calculated time to fully mix when only the molecular diffusion is present (*t*_mix_ = *d*^2^/*D*). The molecular diffusion coefficient D is assumed to be 10^−9^ m^2^/s. The calculated pitch length of the double helix A (design I) is larger when compared to the double helix C (design II). The pitch length can be influenced during fabrication of the thread helices just by twisting the threads in the desired number. By doing so, one is also capable of control the packing of the helices and subsequently the mixing capacity. A tighter thread helix will increase the final touching surface in between the threads. The radii were calculated by using the dimensions of at least three images of cross sections of devices (in duplicate or triplicate). The radii used in all calculations were 1.79 × 10^−4^ ± 1 × 10^−7^ m for the double helix (design I/A), 2.41 × 10^−4^ ± 4.9 × 10^−6^ m for the triple helix (design I/B), 1.77 × 10^−4^ ± 2.4 × 10^−6^ m for double helix (design II/C) and 1.97× 10^−4^ ± 8.5 × 10^−6^ m for triple helix (design III/D).

The radius of curvature of individual channels is larger than the radius of the helix. This can lead to a secondary Dean flow due to the differential flow in channel locations near the center and peripherally, a phenomenon that is quantified by the Dean number.

The curvature ratio λ was the highest at the double helix (I/A) and the lowest for the double helix (I/B). In this work, with consistently low Reynolds numbers as Dean numbers, successful mixing was obtained at small length scales of less than 15 mm despite the fact that the Dean numbers were also quite low. This is supported also by the findings of Nguyen et al. [35] who reported that at low Reynolds numbers of 1 < *Re* < 10, as is the case with our experiments, Dean numbers in this low range are still capable of convective mixing.

**Figure 11:**
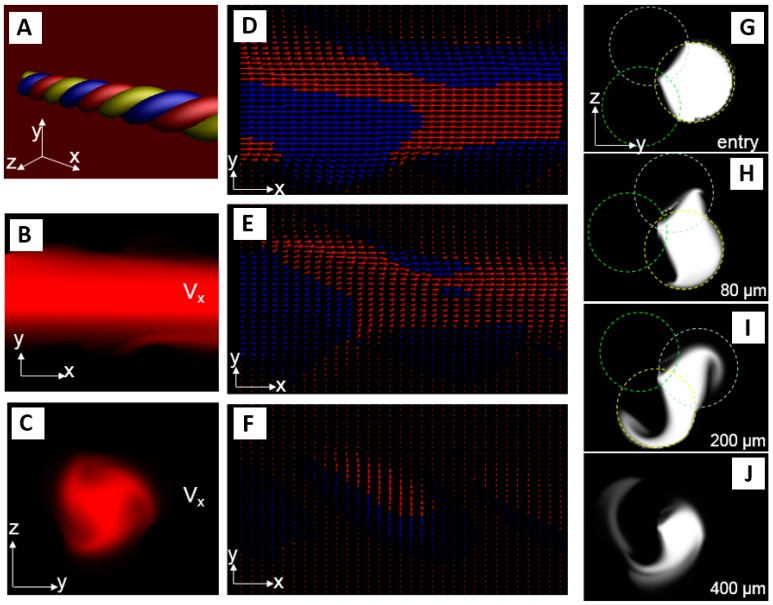
Solid modeling (**A**) and computational fluid dynamics (**B**–**J**) of triple helix channel (thread diameter = 160 µm, driving pressure = 1.25 kPa/mm). Velocity component *v_x_* along channel axis is shown in (**B**) (longitudinal cut) and (**C**) (transverse cut). Highest velocities are found in the center, with a non-parabolic flow profile. (**D**–**F**) show flow vectors in longitudinal cuts at the center, partially offset from the center, and in the helix periphery, respectively (with arrows blue: *z*-component > 0, red: *z*-component < 0). Note the increasing Dean flow component (perpendicular to the channel main axis) in the periphery. A solute (diffusion coefficient 9.3 × 10^−9^ m^2^/s) is injected into one channel (**G**) and mixing observed in serial cross-sections (**H**–**J**): note that in addition to the diffusive component seen as intensity gradient, helicity induces an important convective mixing component perpendicular to the channel axis.

Our computer simulations were performed to explore fluid flow geometry and patterns in such helices with the given design parameters. Simulation results are shown in Figure 11. Our results are also supported by results from Kumar et al. who used computational fluid dynamics to investigate the mixing efficiency of curved tubes and reported that mixing efficiency is a rather complicated function—e.g., the curvature ratio, the pitch and the Reynolds number [14]—and are critically dependent on the curvature ratio, a parameter that can be nicely controlled in our approach.

### 3.2. Assembly of Nanoparticles

**Figure 12:**
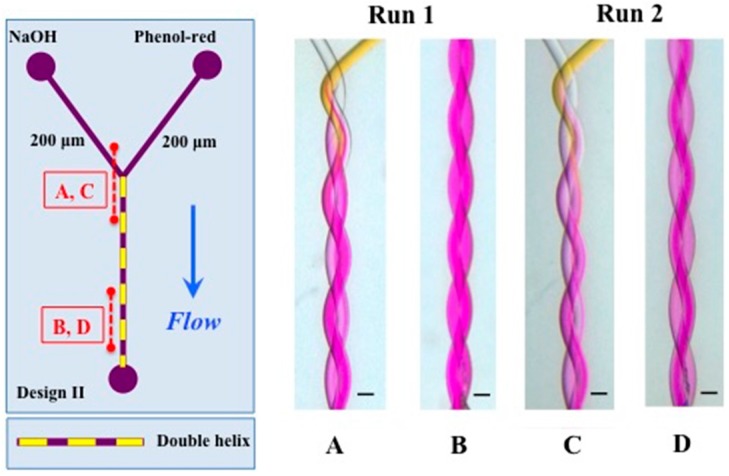
Flow profile in the device used to manufacture the nanoparticles. Run 1 (**A**,**B**) was done with 0.03 mL/h (Phenol-red) and 1 mL/h (NaOH) and for Run 2 (**C**,**D**) the flow rates 1 mL/h (Phenol-red) and 4 mL/h (NaOH) was used. The pitch length is 2.24 mm. The scale bar represents 200 μm.

**Figure 13:**
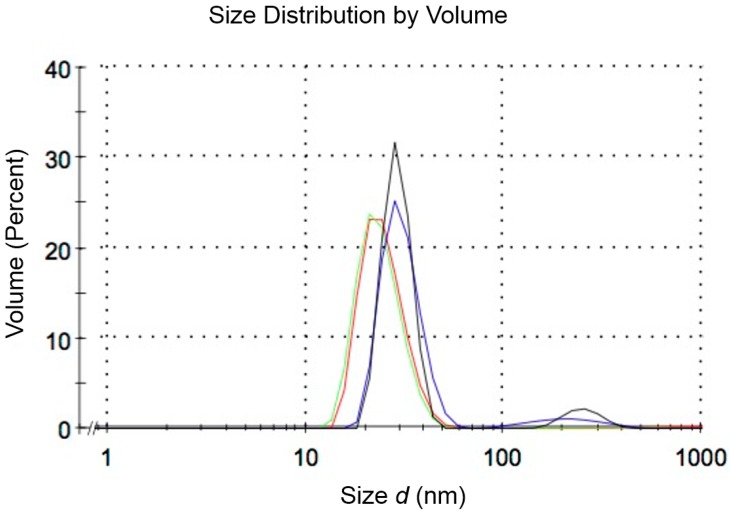
An example of the size distribution obtained by using dynamic light scattering (DLS) on the samples collected in the runs done. In all samples, nanoparticles with a diameter of 24–30 nm were obtained. Each color represents the run of one sample collected (Run 1 (flow rate ratio FRR) = 33.3): **red** and **green** and Run 2 (FRR = 6.8): **black** and **blue**.

In order to test the capability to generate nanomaterials by polymer self-assembly (as reported previously [29]) using helical channels, we studied the self-assembly of an amphiphilic triblock (ABA) copolymer with a double helix system (200 μm inlets; design II in Figure 1). Since microfluidic assembly and nanoprecipitation with these materials mainly depends on the initial concentration of copolymer in the organic solvent, organic phase fraction and flow, two sets (run 1 and 2) with different *Q_T_* and FRR were tested (see Table 1) in a similar way to that done previously [29]. The flow profile inside the device is presented in Figure 12. DLS results are presented in Figure 13, which shows nanoparticles of 29 nm for FRR 6.8 and 24.5 nm for FRR 33.3, with good reproducibility in repeated runs. The output of the device consists almost exclusively of nanoparticles with a narrowly defined size range. Nanoparticle size variation, measured as half-width of the particle size distribution curve in DLS, was 15 nm at FRR = 6.8 and 16 nm at FRR = 33.3. In comparison, using the T-shape device (reported in detail in [29]), self-assembly of the same polymer at FRR = 7 yielded a size of 32.9 nm with a half-width of 26 nm, and at FRR = 30 yielded a size of 24.5 nm with a half-width of 28 nm. Thus, the helical device resulted in comparable nanoparticle size but lower size variation, supporting the benefit of rapid mixing in the new device.

From these results, we conclude that helical microfluidic channels are thus suitable for the in-line, continuous and reproducible assembly of polymer nanoparticles with narrow size variation.

### 3.3. Comparison with Other 3D Mixers

One key differentiating feature of this novel approach to 3D mixing is its rapid prototyping character. True 3D microchannel geometry with good reproducibility and effective control of flow is achieved without the need of cleanroom infrastructure, photolithographic techniques and silicon masters.

In comparison with other microchannels rapid prototyping methods that have been used by our lab, we observe the following:

Compared to 3D printing, we see much higher fidelity and reproducibility of channel geometry with the option of achieving smooth channel surfaces. In addition, the new manufacturing approach leads to much more stable designs that are able to withstand higher driving pressures.

In comparison to laminated PDMS microfluidics, this new approach eliminates a weak point in the microfluidic system structure that required significant attention to avoid leakage or delamination.

In contrast to micromilling, we could achieve significantly smaller features size, much better surface smoothness, and a significantly reduced work effort per device constructed. Also, we found that microdrilling was vulnerable to drill abrasion and required very accurate surface alignment to the machine to achieve reproducible results.

In terms of flow characteristics, we note that this new design impacts flow directionality at all fluid–wall interfaces, in contrast to 2.5D approaches such as herringbone structures that only impact flow direction on one wall. In the latter, in particular at very low Reynolds numbers, two different flow regimes may be encountered with an almost stagnant flow in the recesses of the herringbone, and a near-straight flow above the herringbone, with little curl. Effectively injecting momentum in the direction perpendicular to the main flow axis is of particular importance in this otherwise heavily viscosity-driven flow regime. In comparison to experimental asymmetric, multistage herringbone architectures [36], mixing length is competitive in our design, but we suspect that further design optimizations are conceivable, which is a topic of our ongoing research.

In comparison with some other design/manufacture methods that require multiple manufacturing steps, e.g., manufacture of several layers/parts followed by assembly needing perfect alignment, this new approach allows single-step construction without the need for alignment and assembly.

## 4. Conclusions

The 3D template casting method [20] can be utilized for rapid prototyping of 3D helical microchannels designs which are suited as mixers and as chemical reaction compartments for multi-component chemical reactions, as well as for controlled nanomaterial assembly. Mixing characteristics are advantageous compared to conventional T-shaped mixers because the helix structure combines the diffusive mixing regime in small channels with a convective mixing regime induced by the 3D helix structure. In contrast to established 2.5-dimensional mixing structures such as herring bone channels that are produced by photolithography, the rapid prototyping method reduces the infrastructure requirement and the production time for such systems. Experimental validation reports the utility of such helix designs for 2- and 3-component microfluidic chemistry also for controlled self-assembly of co-polymeric nanomaterials. Future study will explore the realm of applications for such 3D helices and will deepen the understanding of their performance compared to herringbone and other mixer types.

## Figures and Tables

**Table 1:** Two independent runs were done with different flow rates and flow rate ratios.

Run	1	2
*Q*_aq_ (mL/h)	1.00	4.00
*Q*_org_ (mL/h)	0.03	0.59
*Q_T_* (mL/h)	1.03	4.59
FRR	33.30	6.78
%EtOH	3.00	15.00

**Table 2:** Characteristics of the helices used.

Type Helix	*d*_channels_ (μm)	*R* (m)	*R*_curvature_ (m)	λ	*L*_pitch_ (m)	*T*_mix_ (s)
Double helix (I/A)	200/200	1.79 × 10^−4^	1.03 × 10^−3^	5.73	2.44 × 10^−3^	128
Triple helix (I /B)	180/200/200	2.41 × 10^−4^	1.07 × 10^−3^	4.41	2.80 × 10^−3^	232
Double helix (II /C)	200/200	1.77 × 10^−4^	8.94× 10^−4^	5.04	2.24 × 10^−3^	126
Triple helix (III /D)	120/180/200	1.97 × 10^−4^	1.00 × 10^−3^	5.10	2.50 × 10^−3^	155
